# Anesthetic management of a patient with severe aortic regurgitation undergoing reoperation for ascending aorta false aneurysm using hypothermia: prevention of ventricular fibrillation by nifekalant

**DOI:** 10.1186/s40981-021-00446-8

**Published:** 2021-05-20

**Authors:** Akiko Tomita, Tomoko Fujimoto, Shoko Takada, Yukio Hayashi

**Affiliations:** 1 Anesthesiology Service, Sakurabashi-Watanabe Hospital, Osaka, Japan; 2 Present address: Anesthesiology Service, Osaka City General Hospital, 2-13-22 Miyakojima-hondori, Miyakojima-ku, Osaka, 531-0021 Japan

**Keywords:** Resternotomy, Moderate hypothermia, Ventricular fibrillation, Nifekalant, Aortic regurgitation

## Abstract

**Background:**

To prevent cardiac collapse and to protect cerebral function, hypothermic cardiopulmonary bypass is established before resternotomy. However, ventricular fibrillation under hypothermia facilitates left ventricular distension, which causes irreversible myocardial damage when the patient has aortic regurgitation. We report a case of successful management in preventing ventricular fibrillation under hypothermia by using nifekalant.

**Case presentation:**

A 56-year-old male, who had been performed a David operation, was scheduled for a Bentall operation for a pseudo aortic aneurysm with severe aortic regurgitation. After inducing anesthesia, we administered intravenous nifekalant and a vent tube was inserted into the left ventricle under one-lung ventilation. Extracorporeal circulation was established and resternotomy started after cooling to 27 °C. Although severe bradycardia and QT prolongation were observed, ventricular fibrillation did not occur until aortic cross-clamping.

**Conclusion:**

Combining maintaining cerebral perfusion and avoiding left ventricle distension during hypothermia was successfully managed with nifekalant in our redo cardiac patient with aortic regurgitation.

## Background

Resternotomy in a patient with a previous cardiac surgery has a risk of cardiac collapse because of adhesion between the heart, the blood vessels, and the sternum, potentially leading to inadvertent massive and uncontrollable hemorrhage during sternal reentry and dissection [1]. Since the brain is vulnerable to ischemia following cardiac collapse, closed cardiopulmonary bypass (CPB) under hypothermia control is often performed before sternotomy, as it can reduce cerebral metabolic rate, cerebral oxygen requirement, and production of toxic metabolites [2, 3]. One of the complications associated with hypothermia is fatal arrhythmia such as ventricular fibrillation (VF). Once VF occurs, effective cardiac output is lost and distention of the left ventricle increases. This is particularly relevant in the presence of significant aortic regurgitation (AR). Thus, in order to prevent this complication, it is essential to avoid the onset of VF and to conserve contractility until complete division of the sternum and aortic cross-clamping during hypothermic condition. Herein, we report our successful case using nifekalant, a specific potassium channel blocker, to prevent VF and left ventricle distension during high-risk resternotomy in a patient with aortic regurgitation under hypothermia.

## Case presentation

Written patient consent was obtained, and our institutional ethical committee approved the publication of this case report.

**Fig. 1:**
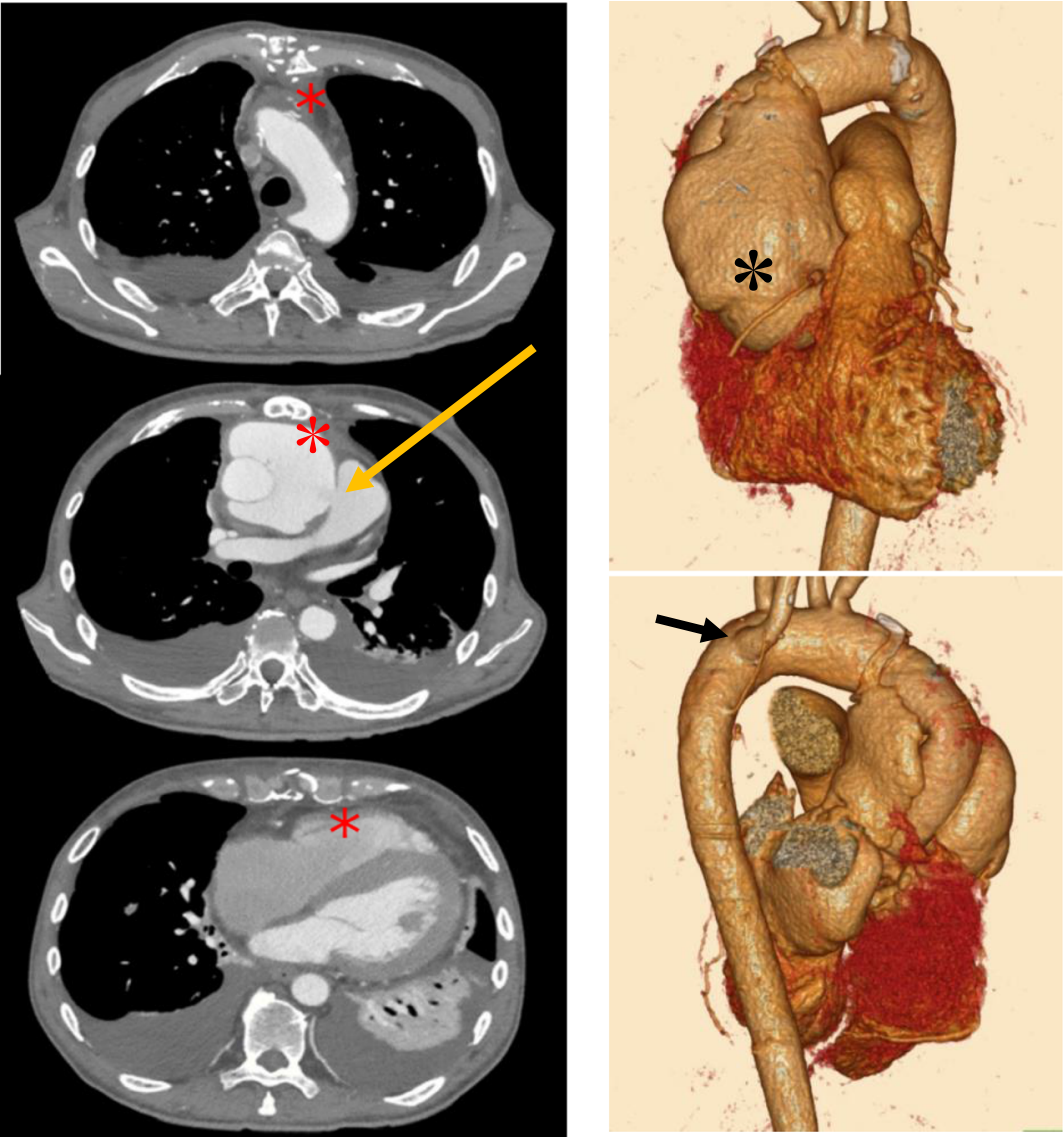
The preoperative images of transverse (left) and three-dimensional (3D) reformatted computed tomography (right). The images showed a large pseudoaneurysm (black asterisk), extensive adhesions between the aneurysm and the sternum (red asterisk), abnormal blood flow from the aneurysm to the main pulmonary artery (yellow arrow), and angiectopia of the right subclavian artery originating from the distal aortic arch (black arrow)

A 56-year-old male (167 cm, 55.6 kg), who had undergone a David operation for type-A acute aortic dissection 7 months ago, was scheduled for a Bentall operation for a pseudo aortic aneurysm and perivascular abscess. The aneurysm had increased from 60 to 76 mm in 3 months before the operation and some blood flow was observed inside the aneurysm. The entry to the aneurysm was identified at the anastomosis with artificial blood vessels above the aortic valve. Blood flow showed a to-and-fro pattern between the left ventricle and the aneurysm. The blood flow from the aneurysm to the left ventricle during diastole was similar to aortic regurgitation (AR), and the degree of regurgitation was moderate to severe. Furthermore, we also found abnormal blood flow from the aneurysm to the main pulmonary artery. Other preoperative transthoracic echocardiography findings included normal systolic function, grade II diastolic function (E/A: pseudo-normalization pattern), mild to moderate tricuspid regurgitation, and moderate pulmonary hypertension. Three months before the operation, he developed heart failure due to perivascular abscess. His preoperative chest X-ray revealed cardiac dilatation (CTR 60%) and bilateral pleural effusion. The electrocardiogram showed sinus tachycardia and complete right bundle branch block. Routine laboratory data were within normal range except hemoglobin 10.4 g dL^−1^, platelet 281,000 μL^−1^, fibrinogen 460 mg dL^−1^, WBC 6200 μL^−1^, CRP 6.78 mg dL^−1^, brain natriuretic peptide (BNP) 1860 pg dL^−1^, and creatinine 1.25 mg dL^−1^. Coronary computed tomography showed no findings suggesting coronary stenosis. Thoracic computed tomography demonstrated the following: extensive adhesion between the aneurysm and the sternum, and angiectopia of the right subclavian artery originating from the distal aortic arch (Fig. 1).

We discussed intraoperative management of the present case at a preoperative conference of anesthesiologists and cardiac surgeons and determined the following algorithm: (1) A left anterolateral small thoracotomy is performed and a left ventricle venting tube is placed to avoid left ventricle distension, because the patient has severe aortic regurgitation. (2) Nifekalant is administered slowly to prevent VF associated with cooling. (3) Institution of full-flow cardiopulmonary bypass (CPB) is done through the femoral vessels or combination of the right axial artery and femoral vessels. (4) Once CPB is established, cooling is initiated by monitoring the bladder temperature and the patient is cooled to 27 °C. (5) At the target bladder temperature of 27 °C, resternotomy is performed. (6) The temperature is maintained until the complete division of the sternum to prevent VF and to conserve contractility. (7) After completing dissection of retrosternal adhesions, aortic cross-clamping is performed, the pseudoaneurysm was incised, and cardioplegia was delivered selectively in the coronary ostia.

General anesthesia was induced with midazolam 5 mg, fentanyl 0.2 mg, and vecuronium 8 mg and maintained with sevoflurane, propofol, remifentanil, and vecuronium. The trachea was intubated with a 37-Fr left double-lumen tube for one-lung ventilation. A percutaneous DC pad was attached on his chest prophylactically. After induction of anesthesia, the left radial and femoral arteries were cannulated for arterial blood pressure monitoring. A central venous catheter and a pulmonary artery catheter were placed through the right internal jugular vein. A transesophageal echocardiography (TEE) probe was inserted for intraoperative cardiac monitoring. Regional cerebral oxygen saturation (rSO2) was measured with near-infrared spectroscopy. We detected the oxygen step-up in the pulmonary artery and calculated that pulmonary flow/systemic flow (Qp/Qs) was 1.7.

**Fig. 2:**
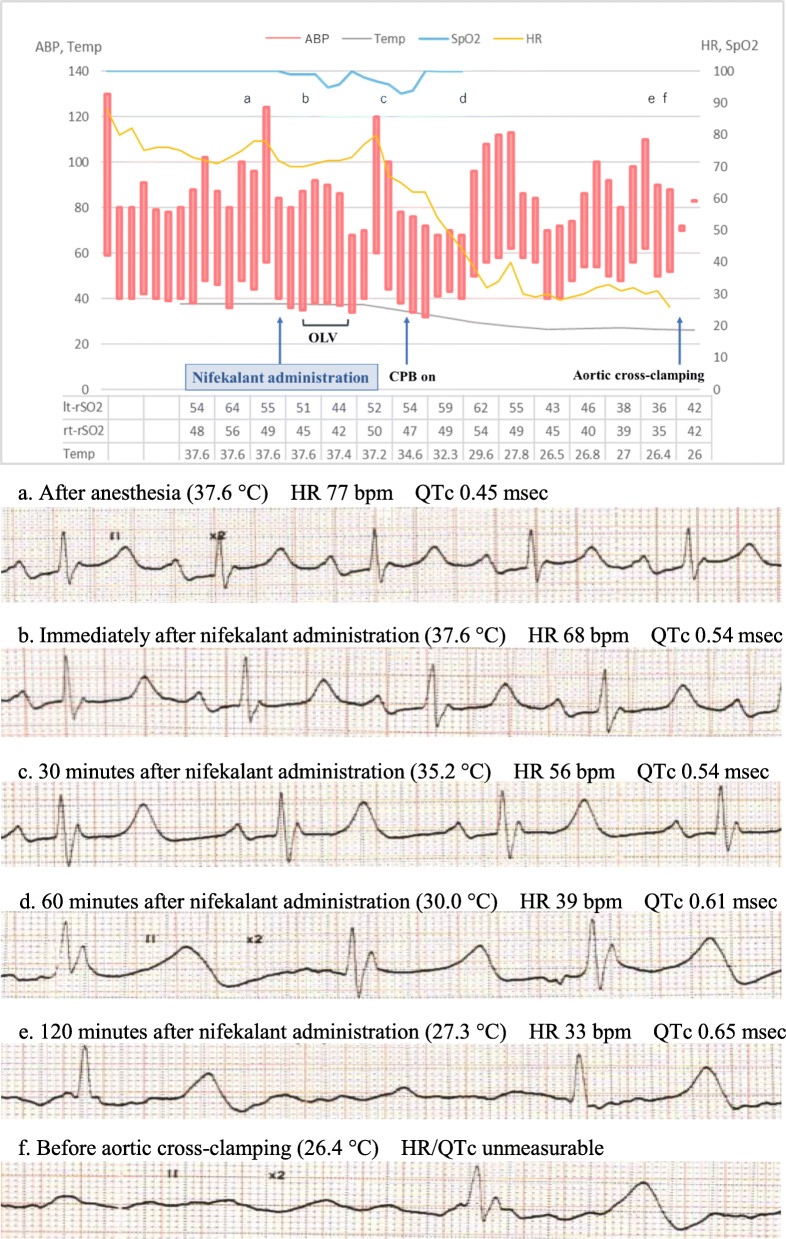
Anesthesia record and electrocardiogram at each indicated point (**a**–**f**) during resternotomy with hypothermia condition. The parentheses show the patient’s bladder temperature. Severe bradycardia, QT prolongation, and premature ventricular contraction were observed before aortic cross-clamping. OLV one-lung ventilation, CPB cardiopulmonary bypass

At first, we administered nifekalant, 10 mg, intravenously. Then, the vent tube was inserted into the left ventricle with a small left thoracotomy under one-lung ventilation. Extracorporeal circulation was established through the cannulation from the right femoral artery and vein, which provided full-flow CPB. Then, cooling was initiated by monitoring the bladder temperature. Resternotomy was initiated after the patient was cooled to 27 °C. The strong adhesion between the sternum and the aortic artery was completely exfoliated without damaging the pseudoaneurysm. Severe bradycardia, QT prolongation, and premature ventricular contraction (PVC) were observed, but VF did not occur under 27 °C (Fig. 2). Bilateral rSO2 decreased with progression of bradycardia (left rSO2 54 to 36% and right rSO2 51 to 35%). Although we increased the blood flow of CPB and transfused red blood cell, the values remained low in 30s. The values continued to be 30s until the aortic clamping and after then improved to 40s. After exposure of the aortic clamp site was completed, we re-started cooling and clamped the aorta at the distal side of the aneurysm and induced asystole by selective cardioplegia. The Bentall operation scheduled was performed without any problem. Weaning from CPB was uneventful and the patient was transported to ICU without extubation. His trachea was extubated on the 5th postoperative day and he had no neurological sequelae.

## Discussion

Pseudo aortic aneurysm is a rare but serious complication following aortic root prosthetic replacement. The present case had severe adhesion between the heart or the blood vessels and the sternum due to redo aortic surgery and he developed severe AR. Therefore, cerebral and myocardial protection are important issues during resternotomy. For this purpose, it is essential to avoid the onset of VF and to conserve contractility under hypothermic condition until complete aortic cross-clamping. In the present case, we administered nifekalant to prevent VF and placed a left ventricle venting tube to avoid left ventricle distension before cooling, leading to successful management.

The mechanism of arrhythmia with hypothermia is considered to be ventricular reentry [4–6]. It is well known that class III antiarrhythmic drugs prolong refractory period, resulting in preventing reentry arrhythmias. Thus, prophylactic administration of nifekalant may be reasonable. Although we chose nifekalant, one may claim that amiodarone can be another candidate for this purpose [7, 8]. There were some reports comparing the two drugs [9–11]. However, the results were controversial, so clear superiority between the two drugs was not concluded. While nifekalant is a pure K channel blocker, amiodarone is a multi-channel blocker including K, Na, and Ca channels and α and β adrenergic receptors. From pharmacological points of view, we considered that amiodarone more strongly suppresses hemodynamics. Furthermore, the blood half-life of nifekalant is shorter than that of amiodarone [12, 13]. In the present case, since we needed the antiarrhythmic effect for only a short duration, that is, from the start of cooling to the aortic cross-clamping, our drug of choice may be justified.

One may claim that hypothermia is known to prolong QT interval, which may induce polymorphic ventricular tachycardia and nifekalant may deteriorate the arrhythmias. We cannot ignore this possibility. To our knowledge, the efficacy of nifekalant in the hypothermic condition has not been reported. In this case, we administered a smaller dose of nifekalant before initiation of cooling. The aim of our idea is to prevent reentry associated with the early stage of hypothermia and to minimize further prolongation of QT interval in later deep hypothermic condition. So far, however, there has been no clear evidence to support or to deny our idea. So, we should prepare the treatment for the arrhythmias, such as percutaneous defibrillation and magnesium administration. The efficacy of nifekalant in hypothermic condition has to be elucidated in the future.

In the present case, bilateral rSO2 decreased with the progression of bradycardia during exfoliating the surgical field following resternotomy, suggesting that cerebral blood flow became inadequate. Thus, we increased the blood flow of CPB and transfused red blood cell, but the value remained low in 30s and was finally improved to 40s by the aortic clamping. Presumably, the reduction of rSO2 seemed to be due to both severe AR and shunt flow from the aneurysm to the main pulmonary artery. Thus, if we had failed to maintain cardiac contractility until aortic cross-clamping, further reduction of the rSO2 values would have occurred. Therefore, nifekalant might indirectly contribute to avoiding postoperative neurological sequelae.

## Conclusion

Combining maintenance of cerebral perfusion and avoidance of left ventricle distension during the cooling phase during resternotomy was successfully managed using nifekalant in our redo cardiac patient with aortic regurgitation undergoing aortic reinterventions.

## Data Availability

The data that support the findings of this report are available from the corresponding author on reasonable reason.
